# Diabetes and Arrhythmias: Pathophysiology, Mechanisms and Therapeutic Outcomes

**DOI:** 10.3389/fphys.2018.01669

**Published:** 2018-11-26

**Authors:** Laurel A. Grisanti

**Affiliations:** Department of Biomedical Sciences, College of Veterinary Medicine, University of Missouri, Columbia, MO, United States

**Keywords:** diabetes mellitus, arrhythmia, atrial fibrillation, cardiac fibrosis, autonomic dysregulation

## Abstract

The prevalence of diabetes is rapidly increasing and closely associated with cardiovascular morbidity and mortality. While the major cardiovascular complication associated with diabetes is coronary artery disease, it is becoming increasingly apparent that diabetes impacts the electrical conduction system in the heart, resulting in atrial fibrillation, and ventricular arrhythmias. The relationship between diabetes and arrhythmias is complex and multifactorial including autonomic dysfunction, atrial and ventricular remodeling and molecular alterations. This review will provide a comprehensive overview of the link between diabetes and arrhythmias with insight into the common molecular mechanisms, structural alterations and therapeutic outcomes.

## Introduction

Diabetes mellitus is a group of metabolic disorders where there are high blood sugar levels over time. Prolonged elevations in sugar levels lead to a number of health complications including cardiovascular disease and kidney disease (Forbes and Cooper, 2013). There are two main forms of diabetes mellitus including type 1, which has an unknown etiology and is characterized by a loss of insulin-producing β-cells in the pancreas resulting in the inability of the pancreas to produce enough insulin (van Belle et al., 2011). Type 1 diabetes is often juvenile in onset, insulin dependent, and comprises roughly 10% of the diabetic patient population. Type 2 diabetes results from insulin resistance and the body's inability to respond to insulin (Kahn et al., 2014). It is generally adult-onset and is a result of genetics and lifestyle choices including excessive body weight, lack of exercise and poor diet. Type 2 diabetes is rapidly increasing in incidence (Centers for Disease C and Prevention, 2008). As of 2015 there were an estimated 415 million people with diabetes worldwide (Federation, 2014).

Cardiac arrhythmia is a group of conditions where the heart beats too fast (tachycardia), too slow (bradycardia) or irregularly (Roberts-Thomson et al., 2011). While most arrhythmias are not serious acutely, prolonged arrhythmic episodes increase an individual's likelihood of stroke, heart failure and cardiac arrest (Nattel et al., 2014). Arrhythmias arise due to a problem in the electrical conduction of the heart however, the cause of these complications is not fully defined. Atrial fibrillation is the most common type of arrhythmia and is associated with significant morbidity and mortality (Kannel et al., 1983). It is becoming increasingly apparent that diabetes mellitus is a significant promoter of cardiac arrhythmias (Kannel et al., 1998).

**Figure 1:**
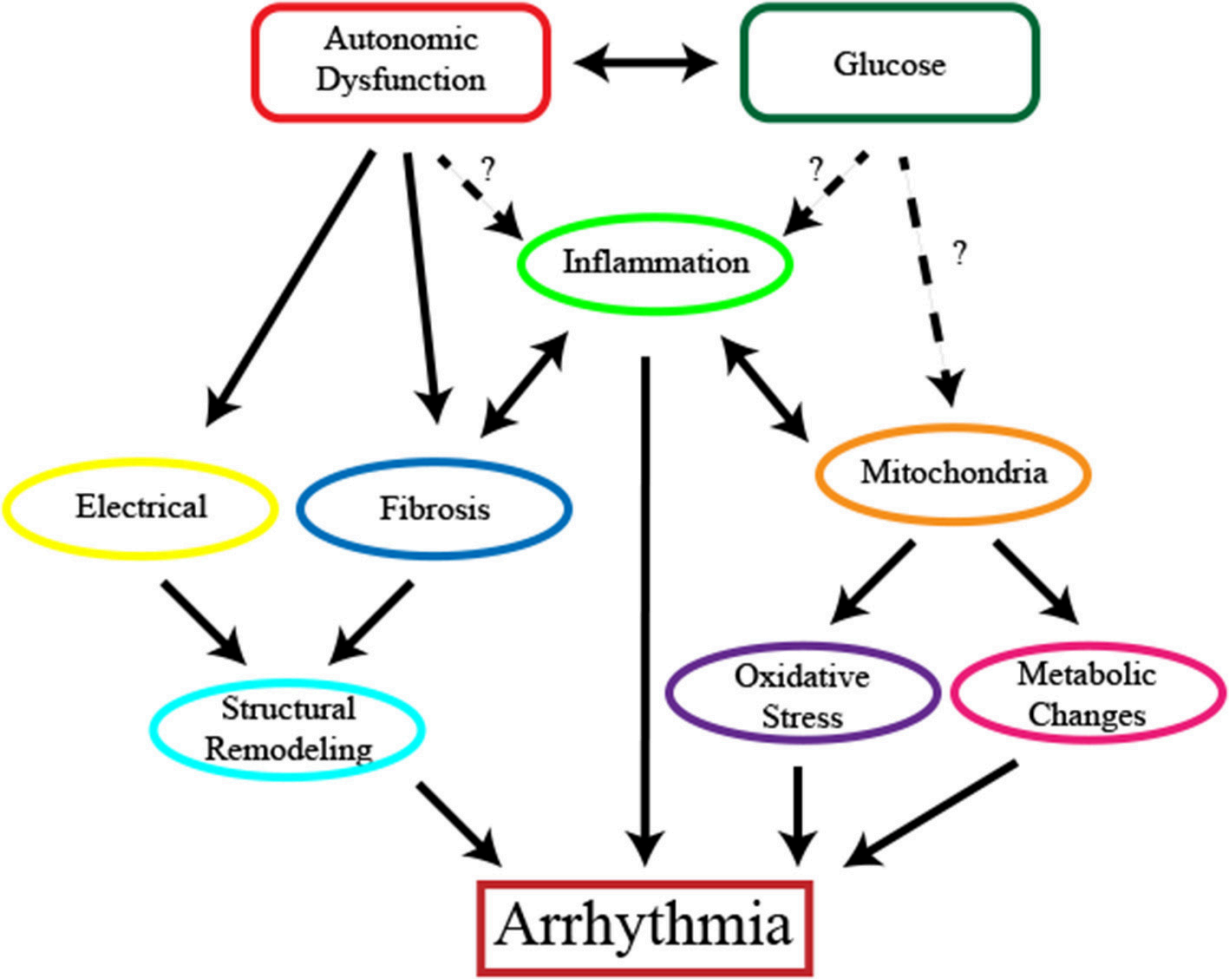
The complex relationship between diabetes and cardiac arrhythmias. Potential contributors to the induction of cardiac arrhythmias including hypoglycemia, hyperglycemia or glucose fluctuations and autonomic dysfunction activate multiple mechanisms to contribute to the development of cardiac arrhythmias. Structural remodeling including changes in the electrical conduction of the heart and fibrosis promote and potentiate the progression of the disease. Mitochondrial dysfunction leads to changes in cardiomyocyte function and metabolism and contributes to disease progression through oxidative stress. Inflammation is present and may arise as a result of oxidative stress and structural changes.

While diabetes likely contributes to multiple types of cardiac arrhythmias, the connection between diabetes and atrial fibrillation has been the most extensively studied to date. Observational studies looking at the association between diabetes mellitus and atrial fibrillation have been inconclusive and inconsistent (Benjamin et al., 1994; Psaty et al., 1997; Wilhelmsen et al., 2001; Nichols et al., 2009; Pallisgaard et al., 2016; Dahlqvist et al., 2017). The incidence of diabetes is most commonly associated with coronary artery disease however, electrical conduction complications are also an important cardiovascular problem associated with both type 1 and type 2 diabetes (Huxley et al., 2011; Dahlqvist et al., 2017). In a 38 year follow up of Framingham heart study patients, diabetes mellitus was identified as an independent risk factor of atrial fibrillation (Benjamin et al., 1994). However, other studies failed to see a connection between atrial fibrillation and diabetes (Wilhelmsen et al., 2001). Discrepancies in these studies may be a result of the populations examined since differences in the association of diabetes and atrial fibrillation appear to be variable depending on age (Pallisgaard et al., 2016), gender (Nichols et al., 2009; Dahlqvist et al., 2017) and ethnicity (Lipworth et al., 2012; Dewland et al., 2013; Rodriguez et al., 2016; O'Neal et al., 2017). In a comprehensive meta-analysis, diabetic patients were found to have an ~40% greater risk for developing atrial fibrillation compared to non-diabetic patients (Huxley et al., 2011) and a more recent meta-analysis, identified a 20% increase in the risk of developing atrial fibrillation for prediabetic patients whereas in patients with diabetes, this number was elevated to 28% greater change of atrial fibrillation development (Aune et al., 2018). Furthermore, this meta-analysis identified a dose dependent relationship between increased blood glucose levels and atrial fibrillation suggesting that rises in glucose may be an important contributor to atrial fibrillation. In a large study, over 845,000 patients, diabetes was found to be a strong, independent risk factor for atrial fibrillation and other cardiovascular diseases mellitus (Movahed et al., 2005). However, obesity, which is common in patients with diabetes mellitus is independently associated with atrial fibrillation and might also be a contributing factor (Grundvold et al., 2015; Baek et al., 2017). Levels of pericardial fat have been linked to atrial fibrillation in humans (Al Chekakie et al., 2010). Though not as extensively characterized, diabetes likely also contributes to ventricular arrhythmias since there is electrocardiographic evidence of this in humans and the underlying mechanisms linking diabetes with atrial fibrillation would apply to other types of arrhythmias (Cardoso et al., 2003). Table 1 summarizes the clinical studies examining the correlation between diabetes and arrhythmias. While there is a clear link between diabetes and cardiac arrhythmias, the mechanisms underlying these changes are not fully elucidated. Potential causes include changes in glucose levels, the autonomic nervous system, structural and electrical remodeling, mitochondrial alterations and inflammation will be reviewed herein (Figure 1).

**Table 1:** Characterization of studies evaluating the correlation between diabetes and arrhythmias. Statistics are reported as [risk ratio (95% confidence interval)].

**Study**	**Location**	**Duration**	**Population characteristics**	**Findings**
Benjamin et al., 1994	United States	38 years	2090 males 2641 females 55-94 years old	Follow-up from the Framingham Heart Study, diabetes was significantly associated with the development of atrial fibrillation (1.4 for men, 1.6 for women)
Dahlqvist et al., 2017	Sweden	10.2 years (non-diabetics)9.7 years (diabetics)	179,980 non-diabetics 35.4 ± 14.5 years old 36,253 type 1 diabetics 35.6 ± 14.6 years old	Slight increased risk in males [1.13 (1.01–1.25)] and greater increased risk [1.50 (1.30–1.72)] in females
Dublin et al., 2010	United States	N/A	2203 control 68 years median age 1410 atrial fibrillation 74 years median age	Increased risk of developing atrial fibrillation in pharmacologically treated diabetic patients [1.40 (1.15–1.71)] compared with control (1.00) whereas non-treated diabetics had no difference [1.04 (0.75–1.45)]
Fatemi et al., 2014	United States and Canada	4.68 years	5042 diabetic-standard glycemic control 5040 diabetic-intensive glycemic control	Intensive glycemic control had no impact on atrial fibrillation incidence compared with standard therapy in diabetic patients
Fontes et al., 2012	United States	~10 years	3023 59.2 ± 6.9 years old	Insulin resistance was no associated with risk of atrial fibrillation
Huxley et al., 2011	Multiple Countries	N/A	1,686,097	Meta-analysis associated diabetes with atrial fibrillation [1.39 (1.10–1.75)]
Huxley et al., 2012	United States	N/A	13,025	Pre-diabetic and untreated diabetes were not associated with increased risk for atrial fibrillation. Type 2 diabetics had an increased risk of atrial fibrillation [1.35 (1.14–1.60)]. No association was observed between fasting glucose or insulin and atrial fibrillation but there was a positive association between HbA1c levels and atrial fibrillation in both diabetic and non-diabetic subjects.
Ko et al., 2018	Korea	8.5 years	1,509,280 30-75 years old	Severe hypoglycemia was associated with increased risk of atrial fibrillation [1.10 (1.01–1.19)]
Lipworth et al., 2012	United States	9 years	3026 white 5810 black >65 years old	Diabetes was associated with an increased risk for atrial fibrillation in both white [1.38 (1.15–1.66)] and black [1.25 (0.98–1.59)] subjects with an elevated incidence in white subjects.
Movahed et al., 2005	United States	10 years	552,624 non-diabetic 293,124 diabetic Primarily male 65 year old average	There was a significant association between type 2 diabetes and development of atrial fibrillation [2.13 (2.10–2.16)] and atrial flutter [2.20 (2.15–2.26)]
Nichols et al., 2009	United States	7.2 years	7159 non-diabetics 10,213 diabetics 58.4 ± 11.5 years old	Positive association of diabetes with atrial fibrillation among women [1.26 (1.08–1.46)] but not men [1.09 (0.96–1.24)]
O'Neal et al., 2017	United States	10 years	8611 white 5077 black 63 year old average	Diabetes was associated with a slightly elevated risk for atrial fibrillation in white subjects [1.21 (1.01–1.45)] but not black subjects [1.06 (0.79–1.43)]
Psaty et al., 1997	United States	3.28 years	4844 combined gender >65 years old	Elevated blood glucose was associated with atrial fibrillation [1.10 (1.04–1.17)]
Pallisgaard et al., 2016	Denmark	16 years	4,827,713 non-diabetics 253,374 diabetics	Diabetes is associated with incidence of atrial fibrillation, particularly in young patients 2.34 relative risk with a 1.52–3.60 (95% confidence level) in 18–39 year olds, 1.52 (1.47–1.56) in 40–64 year olds, 1.20 (1.19–1.23) in 65–74 year olds and 0.99 (0.97–1.01) in 75–100 year olds
Investigators et al., 2013	Multiple Countries	6.2 year median	12,537 50+ years old	Severe hypoglycemia was associated with risk of arrhythmic death [1.77 (1.17–2.67)]
Rodriguez et al., 2016	United States	13.7 years	114,083 non-Hispanic white 11,876 African American 5174 Hispanic 3803 Asian 63 year old average age Females	Diabetes was associated with a slightly elevated risk for atrial fibrillation in women (1.33 for non-Hispanic whites, 1.42 for African American, 1.25 for Hispanic, 1.42 for Asian) with no notable difference dependent on ethnicity
Wilhelmsen et al., 2001	Sweden	25.2 years	7495 males 47-55 years old	No association

## Blood glucose levels

Meta-analysis of clinical populations suggests a dose-dependent relationship between blood glucose levels and atrial fibrillation, implying that glucose levels may be an important contributor to atrial fibrillation onset (Aune et al., 2018). However, this may not be the case since intensive glucose control has not been shown to be beneficial in reducing death from cardiovascular causes or all-cause death in multiple large trials (Group et al., 2008; Duckworth et al., 2009) and has been associated with increased mortality in another (Action to Control Cardiovascular Risk in Diabetes Study et al., 2008). Duration of pharmacological treatment (Dublin et al., 2010) and poorly controlled diabetes have also been linked to increased incidence of atrial fibrillation (Huxley et al., 2012). However, in a prospective study of the Action to Control Cardiovascular Risk in Diabetes (ACCORD) trial cohort of patients, intensive glycemic control did not impact the rate of new-onset atrial fibrillation (Fatemi et al., 2014). Animal studies investigating the involvement diabetes in atrial fibrillation suggest it may be due to glucose fluctuations rather than hyperglycemia (Saito et al., 2014). In a streptozotocin-induced rat model, glucose fluctuations increased incidence of atrial fibrillation, atrial fibrosis, and reactive oxygen species (Saito et al., 2014).

There is increasing evidence that hypoglycemic states may contribute to atrial fibrillation. Several reports of hypoglycemic triggered atrial fibrillations have been reported clinically (Odeh et al., 1990; Celebi et al., 2011; Ko et al., 2018) and information gathered from the Framingham Heart Study suggests insulin resistance does not play a role (Fontes et al., 2012). Severe hypoglycemia in type 2 diabetics was associated with a range of adverse clinical outcomes including death from cardiovascular cause (Chow et al., 2014) and in patients from the Outcomes Reduction with an Initial Glargine Intervention (ORIGIN) trial, severe hypoglycemia was associated with greater risk for all-cause mortality and arrhythmic death (Investigators et al., 2013). In a study using 30 patients with type 2 diabetes and known cardiovascular disease, glucose monitoring in conjunction with electrocardiograms showed patients taking insulin and/or sulfonylurea had a high incidence of severe (< 3.1 mmol/L) but asymptomatic hypoglycemia whereas patients taking metformin and/or dipeptidyl peptidase-4 inhibitors did not and patients with severe hypoglycemia had more ventricular arrhythmias (Stahn et al., 2014). In a separate but similar study, type 2 diabetic patients with a history or risk of cardiovascular disease were monitored for interstitial glucose and ambulatory electrocardiogram simultaneously (Chow et al., 2014). Bradycardia and atrial and ventricular ectopic counts were higher during episodes of nocturnal hypoglycemia further suggesting a role for hypoglycemia in arrhythmic events.

## Autonomic dysfunction

The autonomic nervous system is an important regulator of heart rhythm through innervation by sympathetic and parasympathetic nerves. Dysfunction of the autonomic nervous system is recognized as a risk for development of atrial fibrillation and a contributing factor to disease progression (Agarwal et al., 2017). A link between type 2 diabetes and autonomic dysfunction has also been well established and is recognized as a complication that damages multiple organs including the heart (Mäkimattila et al., 1997; Oberhauser et al., 2001). While the etiology of diabetic autonomic neuropathy is not fully understood, it is thought that metabolic insult, neurovascular insufficiency, autoimmune damage and deficiency in neurohormonal growth factors may be contributing factors to the damage or loss of nerves (Vinik et al., 2003). Despite the fact that cardiovascular autonomic neuropathy has been extensively studied, it remains largely overlooked and serious complication of diabetes (Vinik et al., 2003).

In a study of nearly 2,000 men and women from the Framingham Offspring Study, heart rate variability, an indicator of autonomic nervous system function, was associated with plasma glucose levels and reduced in diabetic and patients with impaired fasting glucose levels (Singh et al., 2000). In healthy, non-diabetic adults, impaired heart rate recovery, another measure of autonomic dysfunction, and a predictor of all cause death in diabetic patients (Wheeler et al., 2002; Cheng et al., 2003), was more common in participants with abnormal fasting plasma glucose levels, which was also true in diabetic patients (Panzer et al., 2002). A closer examination of the link between diabetes and autonomic neuropathy using heart rate recovery as measure of autonomic dysfunction also associated diabetes and autonomic dysfunction with new-onset atrial fibrillation and heart failure independent of other cardiovascular risk factors (Negishi et al., 2013). Interestingly, this study demonstrated an incremental and predictive association between diabetes, heart rate recovery and new-onset atrial fibrillation. There is some evidence that the presence of cardiac autonomic neuropathy in asymptomatic type 1 and type 2 diabetes patients could predict major cardiovascular events including arrhythmias (Valensi et al., 2001). The recurrence of atrial fibrillation is increased in diabetic patients with autonomic neuropathy, which was determined by Ewing's test. Electrocardiographic measurements from diabetic patients with autonomic neuropathy had a longer P-wave duration and dispersion compared to control patients or diabetic patients, suggesting that autonomic neuropathy is causing inhomogeneous atrial depolarization to trigger atrial fibrillation (Bissinger et al., 2011). In a study investigating the changes in autonomic function and repolarization that occurring during prolonged hypoglycemia in type 2 diabetic patients, twelve type 2 diabetic patients and eleven age and body mass index-matched control patients had their glucose levels maintained by hyperinsulinemic clamps at euglycemia (6 mmol/L) or hypoglycemia (2.5 mmol/L; Chow et al., 2017). Differences in autonomic regulation during periods of hypoglycemia, as indicated by heart rate, heart rate variability and blood pressure, occurred between patients with type 2 diabetes and controls during hypoglycemia, suggesting that changes in cardiac autonomic regulation in diabetic patients may occur during hypoglycemic episodes and may contribute to arrhythmias (Chow et al., 2017).

Animal models of diabetes show alterations in cardiac innervation (Gando et al., 1993; Otake et al., 2009; Švíglerová et al., 2011; Thaung et al., 2015). In a streptozotocin-induced diabetic rat model, diabetic rats had increased heterogeneity of sympathetic nerves as measure by immunohistochemistry for tyrosine hydroxylase (sympathetic nerves) and acetylcholinesterase (parasympathetic nerves; Otake et al., 2009). This study also found that sympathetic nerve stimulation increased incidence of atrial fibrillation in diabetic rats. Zucker diabetic fatty rats have elevated resting cardiac sympathetic nerve activity (Thaung et al., 2015). Signs of chronic β-adrenergic stimulation were observed in hearts from these rats including impaired responses to dobutamine stimulation, downregulation of β1-adrenergic receptors and increases in Gαi proteins. These studies demonstrate dysfunction of the autonomic nervous system in diabetes, confirming the findings from human studies.

## Structural remodeling

**Figure 2:**
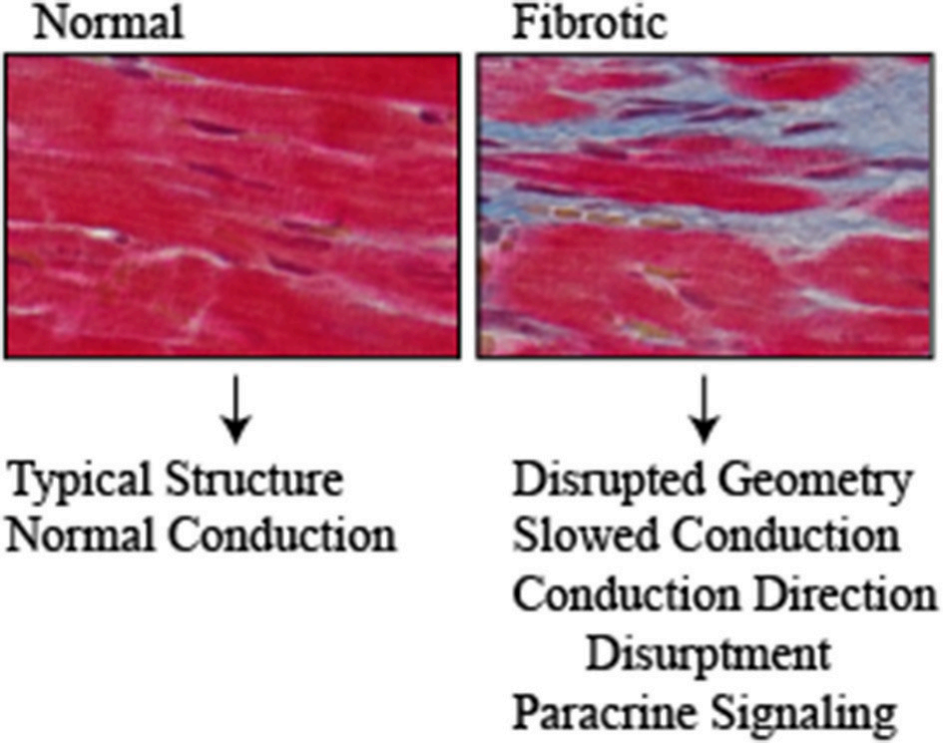
Normal and fibrotic cardiac tissue highlights the structural changes that occur with fibrosis (Red = cardiomyocytes, Blue = fibrosis). Structural changes that occur with diabetes contribute to the pathogenesis of arrhythmias through disrupting the normal architecture of the heart. Fibrosis and fat deposits slow the electrical conduction and disrupt the direction. Furthermore, they serve as a source of paracrine signaling molecules including cytokines/chemokines, adipokines and pro-fibrotic that exasperate the disease.

Structural remodeling likely plays a large role in which diabetes mellitus and obesity promote cardiac arrhythmias. Atrial hypertrophy, fibrosis and fat deposits are observed in the hearts of obese and type II diabetes patients (Tadic and Cuspidi, 2015). Extensive atrial fibrosis is a hallmark of atrial fibrillation and is thought to play a role in both initiating and perpetuation the arrhythmia. Fibrotic tissue in the myocardium disrupts the geometry of the heart and alters the mechanical, electrical and chemical composition (Figure 2). There is extensive evidence of cardiac fibrosis in both type 1 Sutherland et al., 1989 and type 2 diabetes (Regan et al., 1977; Fischer et al., 1984; Nunoda et al., 1985; van Hoeven and Factor, 1990; Shimizu et al., 1993; Kawaguchi et al., 1997) however, the contribution of fibrosis to atrial fibrillation is not fully understood in the context of diabetes. Structural remodeling is particularly relevant in diabetic cardiomyopathy where architectural changes including fibrosis and cardiomyocyte length changes as a result of cardiac dilation increased axial resistance in cardiomyocytes which exasperates conduction dysfunction (Aromolaran and Boutjdir, 2017).

Animal models of diabetes also exhibit signs of increased cardiac fibrosis (Kato et al., 2006; Liu et al., 2012). In Goto-Kakizaki rats, a genetic non-overweight type 2 diabetes model with slight impairments of glucose tolerance, Goto-Kakizaki rats had significantly greater atrial arrhythmogenicity and increased atrial fibrosis compared with control rats (Kato et al., 2006). However, this study did not examine the progression of the disease making it difficult to determine if arrhythmias arose due to atrial fibrosis or if fibrosis is a contributing factor to atrial fibrillation. In a rabbit alloxan-induced diabetic model, diabetic rabbits had increased atrial interstitial fibrosis and electrophysiological changes including a prolonged inter-atrial conduction time and increased atrial effective refractory period which increased the inducibility of atrial fibrillation (Liu et al., 2012).

Advanced glycation end products (AGEs) are proteins or lipids that become glycated as a result of sugar exposure and have become recognized as a major contributor to complications from diabetes (Ramasamy et al., 2011). AGEs and AGE receptors (RAGEs) may contribute to the structural remodeling seen in the diabetic heart. AGEs and RAGEs are increased in streptozotocin-induced diabetic rat atrial and inhibition of AGE formation significantly reduced elevated levels of connective tissue growth factor and atrial fibrosis observed in the diabetic rats (Kato et al., 2008). Elevated RAGE has been associated with atrial fibrillation in humans, however the link between RAGE and diabetes was not observed in this study (Lancefield et al., 2016). However, other mechanisms likely also contribute to the fibrosis seen in the diabetic heart. Studies using fasudil, a Rho-kinase (ROCK) inhibitor, and a high-fat/low dose streptozotocin rat model of diabetes, implemented the RhoA/ROCK pathway in cardiac fibrosis through decreasing RhoA, ROCK and collagen expression (Chen et al., 2014).

While not as extensively investigated as fibrosis, increases in epicardial and pericardial fat is associated with type 2 diabetes (Rosito et al., 2008; Noyes et al., 2014; Levelt et al., 2016) and are correlated with left atrial enlargement, cardiac structural changes, and has been associated with increased atrial fibrillation risk (Al Chekakie et al., 2010; Batal et al., 2010; Wong et al., 2011). Increased epicardial fat is associated with adipocyte infiltration into the myocardium, which contributes to changes in the electrical conduction between cardiomyocytes due to physical disruption and slowing of the conduction time (Friedman et al., 2014; Mahajan et al., 2015; Haemers et al., 2017). Epicardial and pericardial fat is also an abundant source of adipokines and cytokines which have pro-fibrotic and pro-inflammatory effects on the heart. Studies have shown that the secretome from human epicardial fat, including TGF-β family members and matrix metalloproteinases, produces a pro-fibrotic response in rat atrial myocardium (Venteclef et al., 2015). Additionally, pericardial and epicardial fat have increased markers of inflammation including C-reactive protein, IL-6, IL-1β, and TNF-α, which are associated with increased incidence, severity and reoccurrence of atrial fibrillation (Abe et al., 2018). Furthermore, inflammatory mediator production is not reversed by standard therapies including angiotensin converting enzyme (ACE) inhibitors or angiotensin II receptor blockers (Mazurek et al., 2003).

## Role of electrical conduction

Studies consistently demonstrate prolonged action potentials in diabetic patients and animal models. Type 1 (Sivieri et al., 1993) and type 2 (Jermendy et al., 1990; Veglio et al., 2002; Ramirez et al., 2011) diabetic patients have been identified as having slowed conduction velocity and an increased prevalence of prolonged QT interval. In some cases, this has been linked with autonomic neuropathy (Ewing and Neilson, 1990). This is also observed in numerous animal models of type 1 and type 2 diabetes (Xu et al., 2002; Lengyel et al., 2007; Huang et al., 2013). Studies in Goto-Kakizaki (Howarth et al., 2008) and Zucker Diabetic Fatty rats (VanHoose et al., 2010) also identified prolonged QT intervals with additional changes in the R wave amplitudes and signs of autonomic neuropathy. While prolongation of action potentials consistently occurs throughout studies, the mechanisms responsible are less clear. Potassium channels have been the most widely identified change leading to slowed action potentials in diabetic hearts. Prolongation of the QT interval is observed in diet-induced obese mice, which was attributed to a protein kinase D-dependent reduction in voltage gated potassium channel expression (Huang et al., 2013). Oxidative stress-induced alterations in GsH redox state, which will be discussed in more detail in subsequent sections, may regulate K+ channels, that have been shown to be decreased in myocytes isolated from diabetic rats and be reversed by insulin application (Xu et al., 2002). This has also been further supported in follow-up studies, corroborating the involvement of glucose metabolism in these changes (Xu et al., 1996). Changes in outward K+ currents are observed early after streptozotocin injection and corresponded with increases in glucose levels, which were prevented by blocking hyperglycemia (Shimoni et al., 1994). Periodic changes in K+ ion current have been linked to oscillations in energy metabolism in cardiomyocytes, which could be modulated by changing glucose metabolism (O'Rourke et al., 1994). Studies have shown that in cardiomyocytes, glycolysis is more effective than oxidative phosphorylation at regulating K+ channel opening (Weiss and Lamp, 1987). These changes in K+ channels observed in small animals appear to also hold true in larger, more human relevant animal species. In a type 1 diabetes model in dogs, while only slight lengthening in ventricular repolarization was observed, there were decreases in transient outward K+ and slow delayed rectifier potassium currents (Lengyel et al., 2007). In a streptozotocin-induce type 1 diabetes mouse model, prolongation of the QT interval were observed along with increased susceptibility to arrhythmia and decreased K+ currents (Meo et al., 2016). Many of these studies make it difficult to determine the mechanisms responsible for changes in electrical conduction since animal models often have a number of alterations in metabolism including hyperglycemia and hyperlipidemia. However, studies using a cardiac-specific insulin receptor knockout mouse model, several K+ channel components that are important for ventricle repolarization were identified as being decreased, in particular components of the transient outward K+ current fast component, which were also associated with a reduction in the current (Lopez-Izquierdo et al., 2014). Similar with what is seen in other diabetic models and human patients, cardiac-specific insulin receptor knockout mice also had a longer ventricular action potential duration due to a prolonged QT interval, substantiating the role of insulin signaling in diabetes-induced arrhythmias.

Contrarily, not all research implicates K+ currents in action potential changes with diabetes. In a fructose-fat fed rat model of pre-diabetes, QRS prolongation was present, slower conduction velocity, and increased propensity for ventricular fibrillation (Axelsen et al., 2015). There were no changes in Na+ or K+ currents, fibrosis or gap junctions, suggesting another mechanism for dysfunction. In an alloxan-induced diabetic rabbit model, no changes in action potential duration were observed, but there was a reduction in conduction velocity (Stables et al., 2014). A reduction in cell capacitance and Na+ channel density were present in diabetic hearts however, no changes in gap junctions or fibrosis were observed. In obesity-induced QT interval prolongation, there is extensive literature connecting abnormal calcium conduction with arrhythmias (Aromolaran and Boutjdir, 2017). However, this mechanism of arrhythmogenesis is not clear in diabetes and if Na+ or Ca+ channels play an important role in diabetes-induced arrthymias remains to be determined. Energy in the form of ATP is necessary for maintaining membrane potential and generating action potentials, which is mainly supplied by oxidative phosphorylation in the mitochondria, and oxidative phosphorylation to a lesser extent (Barth and Tomaselli, 2009). It is likely that metabolic activity and arrhythmias are interdependent since changes in the cellular energy promotes arrhythmias however arrhythmias also influence metabolic activity. Whole transcriptome analysis of human atrial tissue revealed an upregulation of metabolic process related genes with atrial fibrillation (Barth et al., 2005). This was confirmed in a study using human atrial appendages where metabolomics and proteomics were performed comparing patients with sinus rhythm compared to patients that developed persistent atrial fibrillation following cardiac surgery (Mayr et al., 2008). Patients with atrial fibrillation had an elevation in substrates and enzymes for ketogenic metabolism and other metabolic processes. Additionally, mutantion or knockout of important ion channel genes cause both prolonged ventricular repolarization as well as diabetes (Hu et al., 2014). Hypoglycaemia is also associated with hypokalemia, which could contribute to delayed repolarization (Petersen et al., 1982; Heller and Robinson, 2000; Christensen et al., 2009). As mentioned above, there is extensive clinical and experimental evidence to suggest that structural alterations contribute to the occurrence and persistence of atrial fibrillation (Nattel and Harada, 2014). Changes in gap junctions, which are important for the electrical impulse propagation and synchronization in the heart, are observed with fibrosis and hypertrophy and affect the electrical conduction of the heart (Spach et al., 1988; Saffitz and Kléber, 2004; Ten Tusscher and Panfilov, 2007; dos Santos et al., 2016). In the adult heart, connexin-43 is the main cardiac gap junction component and changes in expression, distribution or post-translational modifications contribute to heart rhythm disturbances (Boengler et al., 2006). Therapies to restore connexin levels are capable of improving conduction disturbances in atrial fibrillation models, further supporting the importance of connexins in the pathogenesis of atrial fibrillation (Igarashi et al., 2012).

There may be alterations in gap junctions in the heart with diabetes. Decreases in phosphorylated and overall connexin-43 levels, a major gap junction protein which has been linked with atrial fibrillation, have been shown in a streptozotocin-induced diabetes model (Mitasíková et al., 2009) which may occur through protein kinase Cε-dependent mechanisms (Lin et al., 2006). These changes were associated with a decreased in connexin-43 phosphorylation and ventricular conduction abnormalities. However, in a different study that also used a streptozotocin-induced diabetes model, connexin-43 levels were elevated and distribution changes were evident (Hage et al., 2017). Other studies using diabetic (db/db) mice show decreased connexin-43 expression that can be reversed with exercise (Veeranki et al., 2016). In this study, exercise resulted in improvements in mitochondrial oxygen consumption rate, tissue ATP levels and reduced cardiac fibrosis with diabetes. Further convoluting the involvement of cardiac connexin-43 in diabetes-induced arrhythmias, an obese diabetic (db/db) mouse model of diabetes showed was atrial hypertrophy and fibrosis without alterations in connexin-43 staining (Hanif et al., 2017). No alterations in connexin-43 levels were also observed in a Zucker Diabetic Fatty rat model of type 2 diabetes, where the conduction velocity was significantly slower in diabetic rats, but levels of connexin-43 were unchanged (Olsen et al., 2013). However, this study did observe distribution changes in connexin-43, which may contribute to functional changes.

Changes in other connexins may also contribute to the pathogenesis of cardiac arrhythmia with diabetes. In a streptozotocin-induced diabetic rat model where connexin-40, 43, and 45 mRNA expression was measured in the sinoatrial node, right ventricle and right atrium, connexin-45 expression was significantly elevated in the sinoatrial node with no changes seen in the atrial or ventricles (Howarth et al., 2007). Using a streptozotocin-induced diabetic rat model, the duration of atrial tachyarrhythmia induced by atrial stimulation was extended in diabetic rats while the conduction velocity was decreased (Watanabe et al., 2012). Increased atrial fibrosis was also observed in diabetic rats compared with controls and had decreased connexin 40 expression with no significant differences in connexin 43. A separate study examining mRNA changes in sinoatrial node of streptozotocin-induced diabetic rats failed to see differences in connexin 40 expression but identified increased transcript expression for connexin 45 among changes in numerous other ion channels including transient receptor potential channel (TRPC) 1, TRPC6, voltage gated calcium channel (Ca_v_) 3.1, Ca_v_β3, ryanodine receptor 3 and Ca_v_γ4 (Ferdous et al., 2016). This same group identified a unique profile of ion channel alterations in the atrioventricular node with no changes in connexins (Howarth et al., 2017).

## Role of mitochondria and oxidative stress

The contribution of oxidative stress to the pathogenesis of cardiac arrhythmias is becoming increasingly recognized (Yang and Dudley, 2013; Samman Tahhan et al., 2017). There are a number of signs of oxidative stress with atrial fibrillation including increased levels of superoxide and hydrogen peroxide (Dudley et al., 2005; Kim et al., 2005; Reilly et al., 2011; Zhang et al., 2012), decreased nitric oxide bioavailability (Cai et al., 2002; Bonilla et al., 2012), changes in the ratio of oxidized glutathione disulfide to reduced glutathione and differences in the ratio of oxidized cysteine to reduced cysteine (Neuman et al., 2007). There is also known to be increased oxidative stress in diabetes, which contributes to the damage of multiple tissue types throughout the body including the heart (Giacco and Brownlee, 2010; Rochette et al., 2014). In diabetes, there is a known increase in superoxide production, which contributes to a reduction in vascular nitric oxide bioactivity through increased NADPH oxidases and dysfunction endothelial nitric oxide synthase (Guzik et al., 2002) which is similar decreases in endothelial nitric oxide synthase and nitric oxide bioavailability are associated with atrial fibrillation (Cai et al., 2002).

Changes in oxidative stress that are present in the heart during diabetes are likely mitochondrial in origin since in diabetic human atrial tissue, where mitochrondrial changes in metabolism of multiple substrates are observed (Anderson et al., 2009). Hydrogen peroxide emissions are increased regardless of the substrate, suggesting alterations in the electron transport system or antioxidant capacity (Anderson et al., 2009). In permeabilized myofibers from right atrial appendages obtained from non-diabetic and type 2 diabetic patients, mitochondria from diabetic patients had a decreased capacity for glutamate and fatty acid-supported respiration, increased content of myocardial triglycerides and increased mitochondrial hydrogen peroxide emission during oxidation of carbohydrate and lipid based substrates (Anderson et al., 2009). There is some evidence that oxidative stress contributes to the atrial remodeling and inflammation seen with atrial fibrillation. In a rabbit alloxan-induced diabetes model, Langendorff perfused diabetic hearts had greater induction of atrial fibrillation following burst pacing, which was decreased with use of the antioxidant probucol (Fu et al., 2015). Antioxidant administration also attenuated atrial interstitial fibrosis and signs of decreased oxidative stress including reductions in serum and tissue malonaldehyde, NF-κB, TGF-β, and TNF-α. However, several studies in humans have shown little or no cardiovascular benefits from antioxidant supplementation demonstrating the need for better understanding of the mechanisms that oxidative stress contributes to atrial fibrillation in the context of diabetes (Sesso et al., 2008; Violi et al., 2014).

There has been limited investigation into the mechanisms linking oxidative stress, diabetes and arrhythmias. One of the most extensively studied mechanisms is Ca^2+^/calmodulin-dependent protein kinase II (CaMKII). CaMKII is a serine-threonine kinase that has emerged as an important nodal point to allow cardiomyocytes to respond to perturbances in calcium and reactive oxygen species through the activation of a diverse group of downstream targets to regulate membrane excitability and calcium cycling (Voigt et al., 2012; Mesubi and Anderson, 2016). CaMKII is increased in atria of atrial fibrillation patients and in mouse models of susceptible to atrial fibrillation (Purohit et al., 2013). CaMKII has been shown to influence calcium dynamics through several different mechanisms in the diabetic heart. In obese Zucker rats and high-fat-fed rodents, there is increased muscle mitochondrial content and CaMKII activation (Jain et al., 2014). Increases in mitochondrial reactive oxygen species and S-nitrosylation of the ryanodine receptor lead to increased SR calcium leak and activation of CAMKII. CaMKII may also contribute to connexin alterations and electrical conduction changes observed in diabetes (Zhong et al., 2017). In ApoE knockout mice fed a high fat diet, downregulation of the ion channels, connexin-43 upregulation and ventricular remodeling could be prevented by administration of a CaMKII antagonist.

Post-translational modifications of CamKII may contribute to its role in diabetes-induced arrhythmias. Animal studies show that mitochondria isolated from streptazocin treated rat hearts have increased total *O*-linked *N*-acetylglucosamine (*O*-GlcNAc) and *O*-GlcNAc transferase levels, with *O*-GlcNAc transferase being localized in the mitochondrial matrix as opposed to an inner membrane localization in control rats (Banerjee et al., 2015). Mislocalization of *O*-GlcNAc transferase results in decreased interactions with complex IV of the electron transport chain, resulting in impairments of its activity. Acute hyperglycemia in cardiomyocytes has been shown to result in covalent and persisting modifications of Ca^2+^/calmodulin-dependent protein kinase II (CaMKII) by *O*-GlcNAc, which can also be observed in the heart and brain of diabetic humans and rats (Erickson et al., 2013). *O*-GlcNAc modification of CaMKII leads to increased activation of spontaneous sarcoplasmic reticulum Ca^2+^ release resulting in arrhythmias (Erickson et al., 2013). In diabetic humans and mouse models of diabetes, there are increased levels of oxidized CaMKII (Luo et al., 2013). Oxidized CaMKII has been linked with ventricular arrhythmia (Wang et al., 2018) and atrial fibrillation (Purohit et al., 2013) however, how oxidized CaMKII contributes to atrial fibrillation with diabetes is not currently defined. In addition to changes in CaMKII, abnormal calcium handling has been observed in diabetic hearts however, how this relates to arrhythmogenesis has no yet been investigated (Belke and Dillmann, 2004; Lacombe et al., 2007).

## Role of inflammation

Inflammation has been identified as a risk factor for cardiac arrhythmias due to the increased frequency of incidence following cardiac surgery (Bruins et al., 1997), genetic studies (Gaudino et al., 2003) and increased occurrence during myocarditis (Spodick, 1976). In human patients, C-reactive protein (Aviles et al., 2003) has been associated with incidence of atrial fibrillation and was able to predict future development and polymorphisms in the interleukin-1 family affect risk for atrial fibrillation (Cauci et al., 2010; Gungor et al., 2013). Inflammation has also been suggested as an underlying pathogenic mediator for diabetes. Since it was identified that TNF-α secretion by adipocytes played a role in the body's update of glucose and response to insulin which contributes to the development of diabetes (Hotamisligil et al., 1993), extensive research has been done looking at the role of inflammation in diabetes (Wellen and Hotamisligil, 2005; Calle and Fernandez, 2012).

The connection between inflammation, arrhythmias and diabetes is not currently well characterized and an ongoing area of research however, hypoglycemia, which has been suggested to trigger atrial fibrillation (Odeh et al., 1990; Celebi et al., 2011; Ko et al., 2018), increases markers of inflammation (Investigators et al., 2013). In a recent study, toll-like receptor (TLR) 2 knockout mice have decreased incidence of arrhythmias compared to wild-type mice in a streptozotcin model of diabetes mellitus (Monnerat et al., 2016). This is thought to occur through IL-1β production by macrophages since macrophages from TLR2 knockout animals had lower levels of MCHII^high^ macrophages and NLRP3 inflammasome. IL-1β was decreased in the hearts of TLR2 knockout animals and IL-1β could decreased potassium current and increase calcium sparks in isolated cardiomyocytes. Furthermore, inhibition of the NLRP3 inflammasome or IL-1β reversed diabetes-induced arrhythmias.

## The impact of diabetes therapies on arrhythmias

Current type 2 diabetes therapies aim to treat hyperglycemia to reduce and maintain glucose concentration to normal levels in an effort to prevent the development of complications (Kahn et al., 2014). Since the process through which diabetes causes arrhythmias is not currently know, the impact of current therapies is just beginning to be understood. Studies suggest that merely controlling glucose levels is not beneficial (Group et al., 2008; Duckworth et al., 2009) and potentially detrimental (Action to Control Cardiovascular Risk in Diabetes Study et al., 2008) in controlling cardiovascular complications. This lack of benefit from intense glycemic control includes the rate impact the rate of new-onset atrial fibrillation (Fatemi et al., 2014). However, poorly controlled diabetes has also been linked to increased incidence of atrial fibrillation showing that the role of glycemic control is not fully understood at this time (Huxley et al., 2012).

Metformin is currently the most widely used medication to treat type 2 diabetes and acts to suppress gluconeogenesis thus lowering glucose levels. Metformin has been associated with decreased atrial fibrillation risk compared with diabetic patients not taking medication (Chang et al., 2014). *In vitro* studies using an atrial cell line demonstrated that metformin decreased reactive oxygen species in response to pacing and prevented cardiomyocyte remodeling (Chang et al., 2014). In diabetic Goto-Kakizaki rats, metformin treatment decreased cardiac fibrosis and arrhythmias (Fu et al., 2018). Alterations in small conductance calcium-activated potassium channels were observed in the atria of these animals, which was corrected with metformin treatment, suggesting that metformin may restore the atrial electrophysiology. Metformin has also been shown to prevent high glucose induction of apoptosis, autophagy and connexin-43 downregulation in H9C2 cells, a ventricular myoblast cell line (Wang et al., 2017). However, there have been reported incidences of onset of atrial fibrillation with metformin use in diabetic patients (Boolani et al., 2011), which may be attributed to lactic acidosis, which occurs rarely with metformin treatment (Salpeter et al., 2010).

Thiazolinediones are peroxisome proliferator-activated receptor-γ agonists, which decrease glucose levels by increasing storage of fatty acids in adipocytes, also decrease incidences of atrial fibrillation onset, which might also be influenced by their anti-inflammatory actions (Chao et al., 2012; Pallisgaard et al., 2017; Zhang Z. et al., 2017). However, other studies have shown in patients with coronary disease, thiazolinediones have no improvements in atrial fibrillation compared with other diabetes treatments including metformin, insulin, sulfonylurea or meglitinides, suggesting that the anti-inflammatory effects of thiazolinediones does not further improve anti-arrhythmia effects of controlling glucose levels (Pallisgaard et al., 2018).

Dipeptidyl peptidase-4 (DPP-4) inhibitors, such as alogliptin, which increases incretin levels to inhibit glucagon release leading to increased insulin secretion are also a common treatment for type 2 diabetes. In an alloxan-induced rabbit model of diabetes mellitus, diabetic rabbits had increased left ventricular hypertrophy and left atrial dilation (Zhang X. et al., 2017). Diabetic hearts had a higher level of atrial fibrillation inducibility and treatment with alogliptin prevented morphological changes and increased propensity for atrial fibrillation. Additionally reactive oxygen species, mitochondrial membrane depolariazation and mitochondrial biogenesis were improved with alogliptin. In a Taiwanese population, DPP-4 inhibitors, in conjunction with metformin, decreased the onset of atrial fibrillation compared to diabetics taking metformin and other second-line therapies (Chang et al., 2017). Other standard diabetes therapies may also have beneficial effects on cardiac arrhythmias. Retrospective studies suggest that there may be differences in sudden cardiac arrest and ventricular arrhythmias between types of sulonylurea medications, where glyburide was found to have lower risk for sudden cardiac arrest and ventricular arrhythmias compared with glipizide (Leonard et al., 2018).

## Arrhythmia therapies in diabetic patients

Pharmacological therapies for arrhythmia include agents that control rate and rhythm. Currently, there has been no research examining the efficacy of anti-arrhythmic medications in patients with diabetes (Dobbin et al., 2018). Surgically, catheter ablation is an established therapeutic option for heart rhythm control in drug resistant patients. Catheter ablation has been shown in a large study composed of 1,464 patients to have the same efficacy and safety in diabetes patients as the general population (Anselmino et al., 2015). However, due to the presence of other atrial fibrillation recurrence predictors such as alterations in the electrical and anatomical composition of the atrial myocytes, metabolic alterations and other comorbidities (D'Ascenzo et al., 2013), the need to redo ablation is more common (Chao et al., 2010; Anselmino et al., 2015). However, smaller studies have shown that while ablation is equally effective in diabetic patients, there are increased numbers of thrombotic or hemorrhagic complications (Tang et al., 2006).

Thromboprophylaxis therapies are also commonly used in patients with atrial fibrillation to decrease risk of stroke and other complications. A number of clinical trials investigating the effectiveness of various theromboprophylaxis therapies have included diabetic subpopulations. In the Rivaroxaban Once Daily Oral Direct Factor Xa Inhibition Compared with Vitamin K Antagonism for Prevention of Stroke and Embolism Trial in Atrial Fibrillation (ROCKET-AF) trial, 40% of the patients had diabetes (Patel et al., 2011). The Randomized Evaluation of Long-Term Anticoagulation Therapy (RE-LY) trial comparing warfarin and high dose dibagatran was composed of about 23% diabetic patients (Connolly et al., 2009). The Effective Anticoagulation with Factor Xa Next Generation in Atrial Fibrillation-Thrombolysis in Myocardial Infarction (ENGAGE AF-TIMI) trial had ~36% diabetic participants (Giugliano et al., 2013). No differences were observed between diabetic and non-diabetic patients in any of these studies. The Apixaban for Reduction in Stroke and Other Thromoembolic Events in Atrial Fibrillation (ARISTOTLE) trail, composed of ~25% diabetic patients, found no difference in the primary outcome of decreased stroke and thromboembolic events between diabetic and non-diabetic patients (Granger et al., 2011). However, diabetic patients had an increased risk of bleeding compared with non-diabetic participants. Taken together, these studies suggest that anti-arrhythmic and anti-thromboprophylaxis therapies are effective in diabetic patients with few adverse effects.

The renin-angiotensin system is involved in the genesis of arrhythmias through its impact on structural and electrical remodeling (Iravanian and Dudley, 2008). Therapies targeting this pathway including angiotensin-converting enzyme (ACE) inhibitors and angiotensin-II receptor blockers (ARB) have been hypothesized to be beneficial in preventing atrial fibrillation occurrence and are currently the focus of numerous studies (Iravanian and Dudley, 2008). Activation of the renin-angiotensin system is often associated with diabetes where it is thought to impact the initiation and progression of the disease (Giacchetti et al., 2005). While there have not been direct studies linking the renin-angiotensin system and diabetes-induced arrhythmias, it is likely that they are intertwined since the renin-angiotensin system impacts nearly all contributing factors including cardiac remodeling, electrical remodeling and inflammation (Iravanian and Dudley, 2008). Inhibitors of the renin-angiotensin system have been shown to reduce cardiovascular events, decrease diabetic complications and can reduce incidence of new onset diabetes (Hansson et al., 1999; Heart Outcomes Prevention Evaluation Study, 2000; Brenner et al., 2001; Dahlof, 2002; Bangalore et al., 2011). This has been confirmed in a large clinical trial investigating the effects of the ARB valsartan on diabetes development in patients with impaired glucose tolerance where valsartan was found to reduce incidence of diabetes but did not reduce the rate of cardiovascular events (Group et al., 2010). However, in patients with impaired fasting glucose or impaired glucose tolerance, the ACE inhibitor Ramipril was not able to reduce new incidence of diabetes in patients with impaired fasting glucose but did promote normoglycemia (Investigators et al., 2006). While renin-angiotensin system targeted therapies show promise in reducing incidence of cardiac arrhythmias and diabetes, additional research is necessary to further understand the mechanisms involved and confirm studies performed in small patient populations.

## Conclusions

The impact of diabetes on the electrical conduction of the heart and development of cardiac arrhythmias is becoming increasingly apparent. Due to the complex, multifactorial nature of diabetes, the relationship between diabetes and cardiac arrhythmias is not yet fully understood however, correlations between increased blood glucose levels, glucose fluctuation and hypoglycemia, and arrhythmias have been observed and are a likely initiator of the disease. Autonomic dysfunction, which is known to contribute to diabetic complications in other tissues potentiates disease progression through changes in the heart's energy needs, the production of paracrine signaling factors and alterations in receptors that influence ion channel activity in the heart. Alterations in the architecture of the heart including fibrosis, fat deposition and hypertrophy change the electrical conduction of the heart and disrupt the pattern of the electrical signal. They are also an important source of paracrine factors that enhance disease progression. Taken together, these alterations within the heart change the electrical conduction by regulating ion channels and gap junctions between cardiomyocytes changing the electrical signaling. While anti-arrhythmic therapies appear to be effective in diabetic patients, the effectiveness of diabetes therapeutics on prevention of cardiac arrhythmias is unclear. Due to the complex nature of diabetes and cardiac arrhythmias, further experimental and clinical research is necessary to fully elucidate the relationship between diabetes and arrhythmias in the hope of developing improved therapeutic strategies in the future.

## Author contributions

The author confirms being the sole contributor of this work and has approved it for publication.

### Conflict of interest statement

The author declares that the research was conducted in the absence of any commercial or financial relationships that could be construed as a potential conflict of interest.
